# Clinical, Ovulatory and Histological Effect of a Postnatal Testosterone Treatment in Female Dogs

**DOI:** 10.3390/ani14071049

**Published:** 2024-03-29

**Authors:** Cynthia Marchetti, Mariela Grisolia-Romero, Marcelo Priotto, Marcela Faya, Cristina Gobello

**Affiliations:** 1 Research Institute of Natural Resources & Sustainability José Sanchez Labrador S.J., Catholic University of Córdoba, Córdoba 5000, Argentina; cynthia_marchetti@hotmail.com (C.M.); mari_griso@hotmail.com (M.G.-R.); 2 Consejo Nacional de Investigaciones Científicas y Técnicas, Buenos Aires 2290, Argentina; 3 Small Animal Clinics, Faculty of Agricultural Sciences, Catholic University of Córdoba, Córdoba 5000, Argentina; priottomarcelo54@gmail.com; 4 Center of Reproductive Physiology, Faculty of Veterinary Sciences, National University of La Plata, La Plata 1900, Argentina

**Keywords:** canine, androgen, neonatal, uterus, ovary, bitch, contraception

## Abstract

**Simple Summary:**

Canine overpopulation is a major problem in many countries. It has been described in some female mammalian species that postnatal androgenization causes reproductive abnormalities. The objective of this study was to describe the reproductive effect of postnatal androgens in female dogs for contraceptive purposes. Ten newborn female puppies were administered testosterone (TE; *n* = 5) or placebo (PL; *n* = 5) subcutaneously. The puppies were followed up until puberty when ovulation was tested and then spayed. The genital tracts were grossly and microscopically examined. At puberty, all the females had normal estrous behavior and ovulated without age and body weight differences. All TE puppies presented mild clitoris enlargement. Ovaries did not reveal differences between groups. Conversely, the uterine glands as well as the height of the uterine epithelium were higher in TE. It was concluded that a postnatal dose of testosterone did not affect ovulatory capacity, nor did it cause gonadal alterations, although it caused an increased area of endometrial glands and a higher uterine epithelium.

**Abstract:**

It has been described in some female mammalian species that postnatal androgenization causes reproductive structural and functional abnormalities. The objective of this study was to describe the clinical, ovulatory and genital effects of postnatal androgens in female dogs. Ten newborn female crossbred puppies were randomly assigned to: testosterone enanthate 18 mg/100 g sc (TE; *n* = 5) or placebo sc (PL; *n* = 5). The puppies were physically followed up until puberty when ovulation was tested by serum progesterone. Then, ovariohysterectomies were performed, and genital tracts were grossly and histomorphometrically examined. At puberty, all the females had normal estrous behavior and ovulated without age and body weight differences. All TE puppies presented mild clitoris enlargement. Gross and microscopical ovarian examination did not reveal differences. Conversely, the endometrial area occupied by uterine glands as well as the height of the glandular and luminal epithelium were higher in the TE than in the PL group (<0.01). The height of the endometrium and myometrial thickness did not differ between groups. It was concluded that a supraphysiological postnatal dose of testosterone did not affect ovulatory capacity, nor did it provoke gonadal histological alterations, although it caused an increased area of endometrial glands and a higher uterine epithelium.

## 1. Introduction

Canine overpopulation is a major problem in most countries and although surgical methods of sterilization have been widely applied, the impact on the dog population is insufficient [1]. In developing countries, unwanted puppies are abandoned in the streets, constituting a problem to both the animal and human health. Safe, efficient and user-friendly pharmaceutical options are required to control the dog overpopulation issue. Spaying has limitations as it is expensive and requires a surgical environment as well as a prolonged period of recovery [2]. Moreover, surgical gonadectomy has limited acceptance in some countries [1]. Currently, the options for transient fertility control are based on hormonal protocols.

In mammals, sexual steroids have an organizational role during late pregnancy and early postnatal life [3]. It is after birth when testicular androgens or their absence have major organizational effects in the immature brain, establishing the gonadotropin secretion pattern of an animal [3]. For this purpose, testosterone is aromatized to estrogens [4]. The developing anterior hypothalamus could acquire a cyclic, in the case of females, or non-cyclic, in the case of males, hormonal pattern of secretion for the gonadal axis. Thus, the administration of exogenous androgens during the postnatal sensitive period could disrupt the normal development and cause permanent anovulation in females.

It has been reported in female rats that there exists a postnatal stage of major tissue differentiation during which a single androgen administration modifies both the hypothalamic cyclic gonadotropin liberation and the target tissue response to peak estrogens [5,6,7,8,9,10]. This early androgenic disruption not only affects the GnRH neuronal network but also the whole gonadal axis including its lower components, i.e., the ovaries and uterus. Rats which were administered testosterone on the first postnatal days developed ovarian atrophy [11] and altered uterine adenogenesis [12]. Androgenized neonatal female pigs presented anovulatory infertility [13]. The same protocol provoked anestrus, masculine behavior and abnormal uterine morphology and function in rats [14,15]. Coincidentally, cats subjected to a similar postnatal treatment presented a high percentage of anovulation and endometrial abnormalities [16].

There is only one previous study in dogs on this topic which was carried out 40 years ago by a research group of behavioral psychologists [17]. Five postnatal beagle females were injected with increasing total doses (5 to 20 mg) of testosterone propionate on alternate days for three months along with a control group which did not receive any treatment. All control females had two normal estrous cycles accompanied by ovulation, progesterone (P4) secretion, sexual receptivity and attractiveness to males. Conversely, the five androgen-treated females had clitoral enlargement and one or two non-receptive ovulatory cycles accompanied by P4 secretion during the 18 months of follow-up. Finally, three out of five bitches apparently developed delayed anovulation as evidenced by their excised ovaries at the end of the study [17].

Considering the previously described effects of postnatal androgens, this treatment could offer a tool for contraceptive strategies in canids. Thus, the aim of this study was to assess the clinical, ovulatory and genital effects of postnatal androgens in female dogs. For this purpose, testosterone enanthate, a potent, slow-release, economically accessible androgen [18] which is available in most countries worldwide, was used as the endocrine disruptor.

## 2. Materials and Methods

### 2.1. Animals and Pharmacological Protocols

Ten newborn female crossbred puppies born in two different litters in our Institutional Dog Colony were included in this study. The puppies were weighed and identified at birth, weaned at 50 days and fed premium commercial puppy food and water ad libitum. This study was reviewed and approved by the Animal Care and Use Committee of the Veterinary School of the Catholic University of Córdoba. All experiments were conducted under the guidelines established in The Guide for The Care and Use of Laboratory Animals, USA.

The puppies were randomly assigned to one of the following treatment groups within the first 24 h of birth: testosterone enanthate 18 mg/100 g (total dose 35–56.5 mg) subcutaneously (Testoviron Depot 250, Bayer, Buenos Aires, Argentina; TE; *n* = 5) or a placebo of 1 mL corn oil subcutaneously (PL; *n* = 5). The pharmaceutical protocol was elected according to a previous study in cats [16] and pilot trials in dogs.

### 2.2. Follow Up

All the puppies were followed up until puberty occurred. Puberty was defined as the appearance of both typical sexual behavior in addition to >80% superficial keratinized vaginal cells on a clean smear background in the vaginal cytology [19]. During the follow-up period, the females were physically examined for estrous signs, e.g., vulvar enlargement, sanguinous vaginal discharge and tail deviation [19]. The appearance of clinical side effects was also recorded during this period.

### 2.3. Vaginal Cytology

From the fourth month of age onwards, vaginal cytology was carried out three times a week [20]. The samples were collected by using a saline-moistened cotton swab. The cotton swab was introduced, directed dorsocranially and rolled gently over the caudodorsal surface of the vagina. After, the swab was removed and rolled onto a glass slide. Color Fast Kit (Biopack, Buenos Aires, Argentina) staining was performed after air drying [20].

### 2.4. Ovulation Diagnosis

Blood samples were collected by peripheral venipuncture fourteen days after the onset of the pubertal estrus. Serum samples were obtained after centrifugation of the whole blood at 3200 rpm (600 g) for 15 min at 4 °C. Ovulation was confirmed by P4 > 5 ng/mL (Elecsys Progesterone II, Diagnósticos Roche, Mannheim, Germany).

### 2.5. Ovariohysterectomy

Once pubertal estrus ended, ovariohysterectomies were performed. The animals were pre-medicated with medetomidine 5 μg/kg, subcutaneously (Detor^®^, Richmond, Buenos Aires, Argentina). Anesthesia was induced with ketamine 2 mg/kg (Ketamina 50^®^, Holliday, Buenos Aires, Argentina) and propofol (Propovet^®^, Richmond Vet Pharma, Buenos Aires, Argentina) 2 mg/kg intravenously. The bitches were endotracheally intubated and then anesthesia was continued using isoflurane and oxygen in a closed system and intravenous remifentanil 5 μg/kg/h (Remifentanilo^®^, Richet, Buenos Aires, Argentina) as an analgesic during surgery. Ovaries and uteri were excised after mid-line laparotomy was performed [21]. After surgery, meloxicam 0.1 mg/kg was injected subcutaneously (once) and then orally every 24 h for 4 additional days (Meloxivet^®^, Jonh Martin; Buenos Aires, Argentina), and cephalexin 15 mg/kg was administered orally every 12 h for 7 days (Cephilexine^®^, John Martin, Buenos Aires, Argentina). All the bitches were then placed for responsible adoption.

### 2.6. Gross and Histomorphometric Examinations

Ovaries were measured (cm) and weighed (g), then the volume and gonadosomatic index (%) were calculated. The gonads were then cut longitudinally, introduced into Bouin’s fixative for 12 h, then transferred to 70% alcohol and processed routinely with paraffin embedding. Then, 10 μm serial sections were sectioned, mounted on slides, dried, deparaffinized in xylene, rehydrated in graded ethanol solutions and 20 equidistant sections were stained with hematoxylin and eosin (Biopack, CABA, Buenos Aires, Argentina) to be assessed. Ovarian follicles were classified into five groups based on morphology and the number of follicular cells in the widest cross-section [22]: (1) primordial (an oocyte without a zona pellucida (ZP) surrounded by a single layer of flattened granulosa cells; (2) primary (oocyte with distinctive ZP surrounded by a single layer of cuboidal granulosa cells); (3) secondary (oocyte surrounded by several layers of granulosa cells); (4) early antral (space among granulosa cells or a segmented cavity with two or more compartments); (5) antral (one large, continuous cavity) or atretic (degenerated granulosa cells and follicular fluid containing cellular debris). Corpora lutea were also recorded. The total number of each follicle type was calculated following the Gougeon and Chainy [23] formula: Nt = No × St × ts/So × do, where No = number of follicles observed; St = total number of sections; ts = width of the sections (μm); So = sections observed; and do = mean diameter of the nucleus of follicle type. Only follicles with a complete visible oocyte nucleolus were counted. The relative proportion (%) of each follicle type was also assessed.

Uteri cross-sections (approximately 0.5–1 cm) were obtained between the external uterine bifurcation and the tip of each horn and processed as explained for the ovaries. The area occupied by uterine glands per μm^2^ of endometrium over the total area of each microscope field was measured by planimetry. The height of the glandular and luminal uterine epithelium was assessed by counting 100 cells in a total of 10 images per uterus taken with a 10X objective. The endometrial and myometrial thickness were examined in a total of four images per uterus taken with a 4X objective (Image J, V 1.8.0, NIH, Bethesda, MD, USA). All histological images were obtained using a microscope (Olympus BX50, Tokyo, Japan; 10×) through an attached digital RGB video camera (Evolution VF Color, Q Imaging, Tucson, AZ, USA) and digitalized in a 24-bit true color TIFF format. Finally, the images were analyzed using Image Pro Plus v6.0-Media Cybernetics (Silver Spring, MA, USA).

### 2.7. Statistical Analysis

Qualitative and quantitative differences between TE and PL groups were conducted by the Student t and Fisher exact tests, respectively. All the variables were expressed as the mean ± SEM and the level of significance was set at *p* < 0.05 (SPSS 18.0, SPSS, Chicago, IL, USA).

## 3. Results

**Figure 1:**
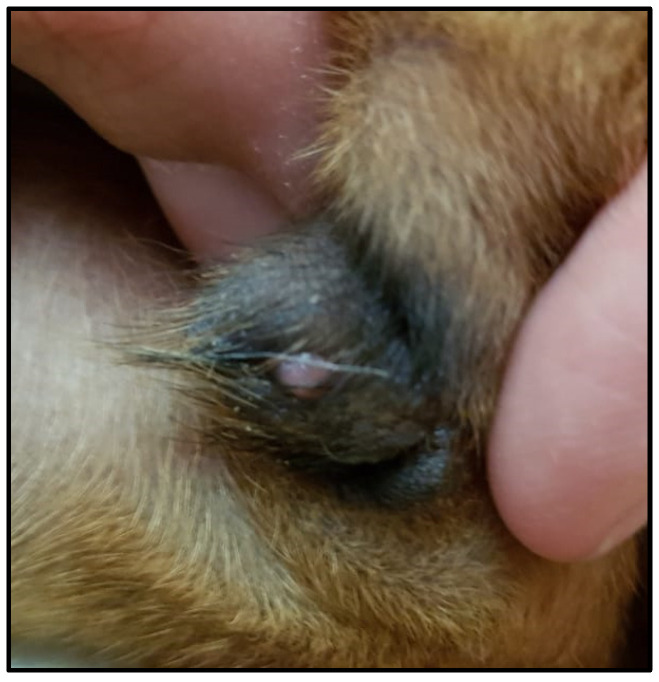
Mild clitoris enlargement in one of the bitches treated postnatally with testosterone enanthate in Table 1.

Age and body weight at puberty attainment did not differ between the TE and PL groups (Table 1). All TE but none of the PL puppies presented mild clitoris enlargement (Figure 1). At puberty, all the females of both groups had normal estrous behavior and ovulated.

Gross ovarian examination revealed corpora lutea in all the TE and PL bitches without differences in any macroscopic variables (Table 1). Microscopically, neither the ovarian follicle numbers nor their relative proportion differed between groups (Table 2).

**Figure 2:**
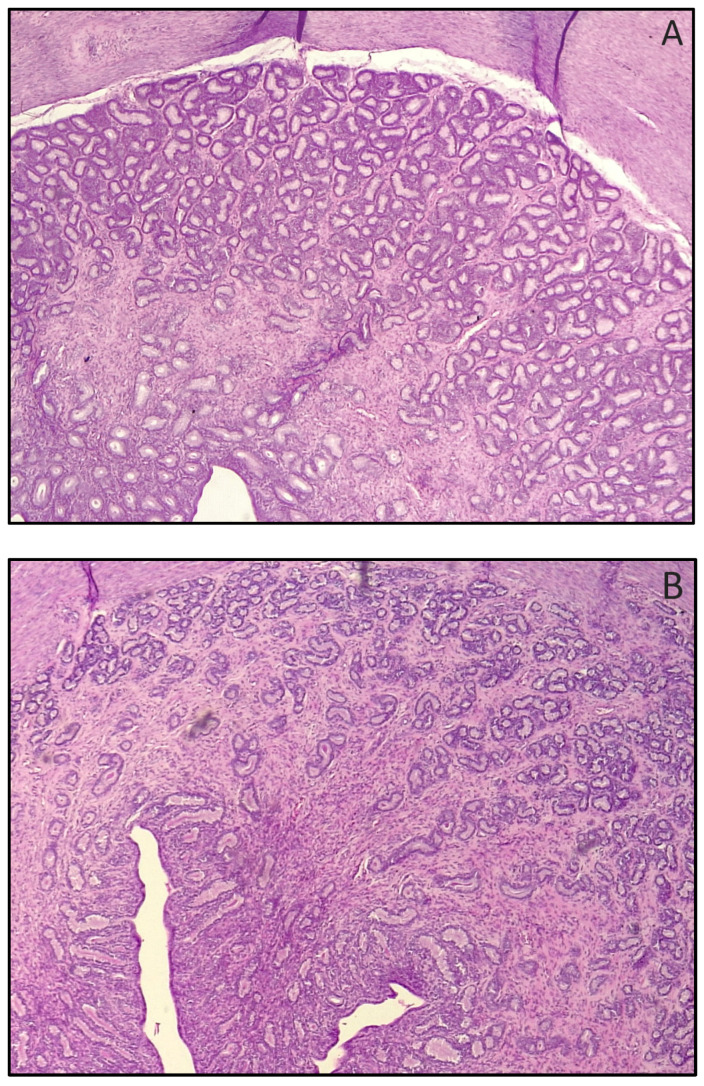
Endometrial area occupied by uterine glands in testosterone (**A**) and control (**B**) treated bitches in Table 1 (H&E, 10×). Notice the higher thickness and density in (**A**) (*p* < 0.01).

Uterine microscopic evaluation revealed that the endometrial area occupied by uterine glands as well as the height of the glandular and luminal epithelium were higher in the TE than in the PL group (Table 3; Figure 2). Conversely, the height of the endometrium and myometrial thickness did not differ between the two groups (Table 3).

## 4. Discussion

To test the hypothesis that in domestic dogs, like other mammalian species [13,17,24], exogenous androgens induce anovulation and genital abnormalities if administered during the postnatal critical developmental period, a single supraphysiological postnatal dose of a long-term release testosterone compound was administered to female puppies.

Evidently, this pharmacological protocol did not suppress the female neuroendocrine regulation type in these pubertal canids. The previous dog study used a decreasing dose that ranged from 0.017 to 0.0035 mg/g of body weight of a short-term release (propionate) testosterone formulation on alternate days for 3 months, apparently obtaining delayed anovulation in 60% of the animals and 100% clitoral hypertrophy [17]. Unfortunately, the use of a short-term androgen in that study makes dose comparisons with the present study difficult. Furthermore, from a practical perspective, prolonged daily administrations as used in that study clearly represent a limitation.

It has been suggested that the degree of hypothalamic alteration produced by androgens is dosage-dependent [5]. Furthermore, it has been shown in rats that insufficiently high perinatal androgen treatments could present a delay in the onset of the anovulatory syndrome until well beyond puberty [24]. Whether anovulation would have developed on successive estrous cycles was not tested in these bitches as it would not represent a practical contraceptive option. Although larger androgen doses might prevent ovulation in this species, side effects will have to be weighed against efficacy. In this study, the undesirable effects physically detected were mild. Conversely, androgen side effects in children include mild to severe virilization, bone mass accrual, stunting of final height, hepatopathy and cholestasis [25]. Care should be taken, as similar abnormalities could appear in puppies administered larger doses.

In order to compare studies, the dose per body weight was calculated for the different species. Recently, a lower dose (0.125 mg/g) of testosterone enanthate caused 75% anovulation in domestic cats [16]. Lower postnatal doses (0.005 mg/g) of the same compound produced 55.6% of anovulation in female mice [26]. An initial study found that a minimal single dose of 0.002 mg/g of testosterone propionate was required to obtain 70% anovulation in rats [27]. Evidently, species differences may also have a role in the final androgen response during this critical postnatal window. Finally, minor variations in the selection of the time window of treatment may also account in the results of the required disruption [8].

In mammals, the earliest stages of folliculogenesis occur during both fetal and neonatal periods under the influence of numerous factors including steroid hormones. The establishment of a pool of primordial follicles during these periods is important for the future reproductive lifespan of a female [28]. Although it has been reported that neonatal androgenization alters ovarian function [29], this alteration in functionality did not seem to be expressed in either gross or microscopical morphology in these females. In line with previous similar studies in laboratory rodents [26,30,31,32], no significant difference was observed in the number of ovarian follicles between the TE and PL.

Although no major difference in uterine structure was discernible between groups, similarly to other reports [12,14,27], the height of the luminal was larger in TE bitches. It has been stated that the functional ovarian abnormality that neonatal androgenization provokes causes high estradiol and testosterone concentrations which, in turn, may induce the proliferation of the superficial epithelium [29]. Furthermore, when flutamide, an antagonist of the androgen receptors, was neonatally administered to laboratory rodents, a decrease in uterine epithelial proliferation was obtained [12]. Whether these changes would be responsible for alterations in the processes of implantation and maintenance of the embryos remains to be unveiled in canine species.

## 5. Conclusions

It was concluded that a supraphysiological postnatal dose of testosterone enanthate did not affect ovulatory capacity, nor did it induce gonadal histological alterations, although it caused an increased area of endometrial glands and a higher uterine epithelium accompanied by minor side effects. In spite of the fact that negative findings are seldom reported, we expect that these results contribute to the knowledge of the limited effect of postnatal testosterone as a contraceptive strategy in canine species. Further research is still guaranteed, as other endocrine disruptors administered during this critical organizational time window could provoke reproductive permanent alternations.

## Figures and Tables

**Table 1:** Ovarian gross parameters (mean ± SEM) of bitches treated postnatally with testosterone enanthate 18 mg/100 g (*n* = 5) or placebo (*n* = 5) and ovariohysterectomized after a pubertal estrous cycle.

	Testosterone	Control	*p*
Age at puberty (weeks)	51.75 ± 9.94	53.50 ± 11.26	>0.05
Body weight (kg)	14.23 ± 4.67	15.50 ± 5.48	>0.05
Ovarian volume (cm^3^)	0.98 ± 0.32	1.08 ± 0.38	>0.05
Ovarian weight (kg)	1.20 ± 0.34	1.32 ± 0.43	>0.05
Gonadosomatic index (%)	0.02 ± 0.00	0.02 ± 0.00	>0.05

**Table 2:** Ovarian follicles (mean ± SEM) and their relative proportion (%) of the bitches in Table 1.

	Treated		Control		*p*
	Mean ± SEM	%	Mean ± SEM	%	
Primordial	72,938.96 ± 13,006.80	87.59	112,323.38 ± 3285.77	91.06	0.08
Primary	3947.38 ± 1155.41	4.74	8275.60 ± 3574.91	6.71	0.49
Secondary	6158.87 ± 3494.90	7.40	2680.62 ± 857.76	2.17	0.44
Antral	226.26 ± 117.58	0.27	68.34 ± 1.53	0.06	0.33
Total	83,271.47		123,347.94		

**Table 3:** Uterine microscopic parameters (mean ± SE) of the bitches in Table 1.

	Treated	Control	*p*
	Mean ± SEM	Mean ± SEM	
Height of the endometrium	925.46 ± 88.56	986.27 ± 74.18	0.41
Area of the uterine glands/μm^2^ of the endometrium	0.35 ± 0.01	0.23 ± 0.01	<0.01
Height of the luminal epithelium	7.63 ± 0.09	6.59 ± 0.05	<0.01
Height of the glandular epithelium	11.11 ± 0.20	8.93 ± 0.12	<0.01
Myometrial thickness	922.51 ± 106.82	903.31 ± 42.10	0.93

## Data Availability

Data are contained within the article.
